# Synovial Cyst of the Atlantoaxial Joint Removed through a Posterior Intradural Approach

**DOI:** 10.1155/2021/9941503

**Published:** 2021-06-03

**Authors:** Atsuhiko Toyoshima, Kiminori Sakurai, Nobuhiro Sasaki, Miyuki Fukuda, Shigeo Ueda, Minoru Houshimaru, Hiroaki Manabe

**Affiliations:** ^1^Shin-Aikai Spine Center, Katano Hospital, Katano City, Osaka, Japan; ^2^Shinagawa Shishokai Hospital, Department of Spinal Surgery, Tokyo, Japan

## Abstract

*Introduction*. Synovial cysts rarely develop in the atlantoaxial joint. We report a case of posterior C1-2 laminectomy for a synovial cyst of the atlantoaxial joint which passed through the dorsal dura and put pressure on the cervical spinal cord. *Case Presentation*. A 62-year-old man with rapid progression of pain and weakness in the left upper extremity presented to our hospital. A cervical spine X-ray showed left C5-6 and C6-7 stenoses. A cervical magnetic resonance imaging showed an intradural extramedullary cystic lesion on the right side of the ventral cervical spinal cord at the C1-2 level and severe compression of the cervical spinal cord. Because a cyst was partially enhancing, a tumor lesion was not identifiable. Due to severe spinal cord compression, we performed intradural cyst removal via a posterior intradural approach with C1-2 laminectomy and left-sided C5-6 and C6-7 foraminotomies. One year after surgery, the cyst did not recur, and atlantoaxial instability did not appear. *Discussion*. A compressive lesion on the cervical spinal cord was not identified preoperatively as a synovial cyst. However, intraoperative and pathological findings suggested that the compressive lesion can be a synovial cyst which passed through the dorsal dura. The surgical treatment strategy for a synovial cyst of the atlantoaxial joint is controversial due to factors, such as the presence of atlantoaxial instability, level of cyst causing compression of the cervical spinal cord, severity of myelopathy, and cyst location. In the present study, the cervical spinal cord was highly compressed and the cyst was located on the right side of the cervical spinal cord; we chose cyst removal through a posterior intradural approach with C1-2 laminectomy.

## 1. Introduction

A synovial cyst of the atlantoaxial joint is relatively rare. Even among reports on a synovial cyst of the atlantoaxial joint without complications of rheumatoid arthritis or other types of connective tissue disorders, a synovial cyst has seldom been found in the atlantoaxial region, and there are not so many reports on this subject. A synovial cyst of the atlantoaxial joint can be found accidentally or detected when cervical myelopathy develops as a result of compression on the cervical spinal cord [1–3]. For treating a synovial cyst of the atlantoaxial joint, surgical procedures include resection via a posterior approach/transoral approach, posterior cervical fusion alone, occipitocervical fusion alone, and combined approach with resection and fusion surgery, and the procedures must be determined based on the size and localization of a synovial cyst [1, 4, 5].

Herein, we report a case of posterior C1-2 laminectomy for a synovial cyst of the atlantoaxial joint which passed through the dorsal dura and put pressure on the cervical spinal cord.

## 2. Case Report

A 62-year-old man had chief complaints of pain in the area between the left axilla and upper extremity and weakness of the left upper extremity. The patient had a medical history of pneumonia and enlarged prostate. Two weeks before his first visit, he noticed pain in the area from the left axilla to the left upper extremity. Then, one week later, he developed left upper extremity weakness and presented to our hospital.

In a physical examination, the patient complained of neuralgia in the area from the left axilla to the ulnar aspect of the left upper extremity. Results were as follows: grip strength (right 33 kg; left 15 kg; a left-hander), left brachioradialis (4/5 manual muscle testing (MMT)), and pronator teres muscle/supinator muscle and extensor digitorum muscle in the left forearm (2/5 MMT), showing a decrease in the left upper extremity. Impairment of skillful movements occurred in the left hand. In tendon reflex, reduced reflexes on left bicep and left brachioradialis were observed, and reflex on bilateral patellar tendon was enhanced.

According to hematological and biochemical findings, a blood test did not show any abnormality, and tumor marker levels were not elevated.

**Figure 1:**
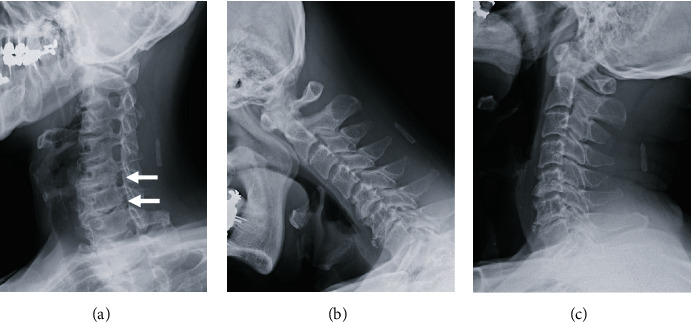
Cervical spine X-ray images. The left oblique view (a) revealed the left C5-6 and C6-7 stenoses (arrow). Anteflexion (b) and retroflexion (c) did not show cervical spine instability.

A cervical spine X-ray revealed the left C5-6 and C6-7 stenoses, but a kymograph did not show cervical spine instability (Figure 1).

**Figure 2:**
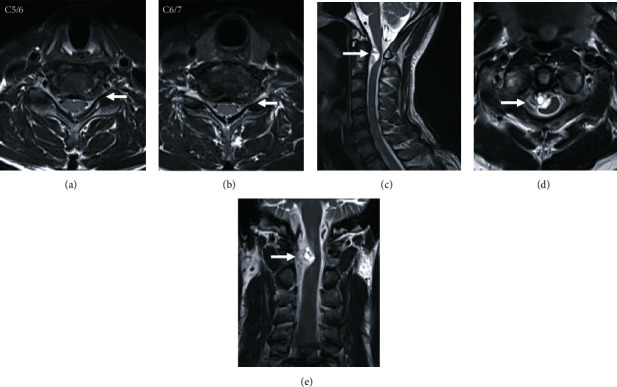
Noncontrast-enhanced magnetic resonance images. In horizontal sections (a, b), cervical disc herniation happened in the same region as the left C5-6 and C6-7 stenoses (arrow). At the C1-2 level, an intradural extramedullary cystic lesion existed in the right side of ventral cervical spinal cord, which severely compressed the spinal cord ((c) sagittal, (d) horizontal, and (e) coronal, arrow).

In addition to the left C5-6 and C6-7 stenoses, left C5-6 disc herniation was seen on a cervical magnetic resonance imaging (MRI) scan. Furthermore, an intradural extramedullary cystic lesion on the right side of ventral cervical spinal cord at the C1-2 level severely compressed the spinal cord (Figure 2).

**Figure 3:**
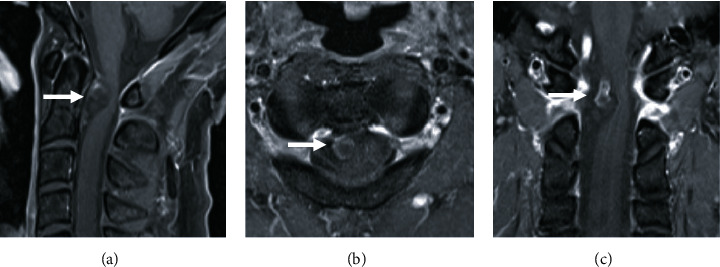
Contrast-enhanced magnetic resonance imaging of the cervical spine. Because a cyst was partially enhancing, a tumor lesion was not identifiable ((a) sagittal, (b) horizontal, (c) coronal, arrow).

**Figure 4:**
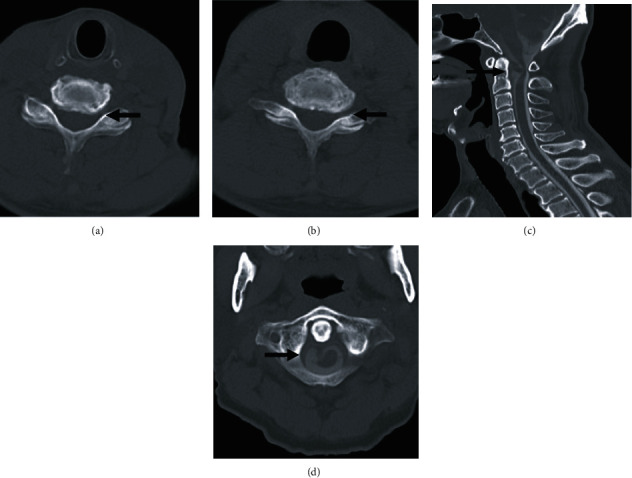
Noncontrast-enhanced computed tomography (CT) and CT myelography. Foraminal stenosis of the left C6-7 intervertebral foramen was identified in the horizontal section ((a, b), arrow). CT myelography showed an intradural extramedullary cyst ((c) sagittal; (d) horizontal, arrow).

A cyst was partially enhancing in contrast-enhanced MRI, and a tumor lesion was, therefore, not identifiable (Figure 3). Contrast-enhanced computed tomography of the spinal cord showed foraminal stenosis of the left C6-7 intervertebral foramen, along with an intradural extramedullary cyst (Figure 4).

On the basis of physical examination and imaging findings, left C5-6 disc herniation, left C6 and left C7 radiculopathy caused by the left C6-7 stenosis, and cervical myelopathy caused by an intradural extramedullary cystic lesion at the C1-2 level were identified.

**Figure 5:**
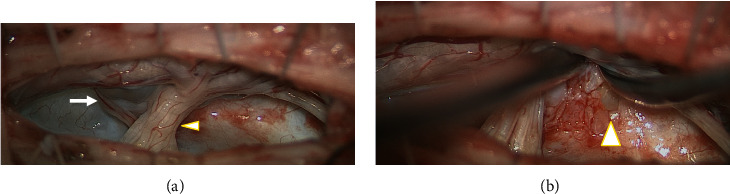
Intraoperative findings. When the dura mater was incised at the C1-2 level, followed by incision in the denticulate ligaments on the right side of the spinal cord, a cystic lesion was present in the right C2 ventral root (arrowhead). The cyst (arrow), which caused compression and significant displacement of the cervical spinal cord from the right ventral side (a), was removed. The cyst capsule, however, seemed to remain in the ventral side and extend posterior to the odontoid process. We found that the dura mater was partly missing in the ventral side ((b) partially missing dura matter: arrowhead).

Because the cyst severely compressed the cervical spinal cord and was located on its right side, we performed a cyst removal through posterior C1-2 laminectomy and left-sided C5-6 and C6-7 foraminotomies. After dura mater incision at the C1-2 level, the denticulate ligaments situated on the right side of the spinal cord were incised. Then, an intradural extramedullary cystic lesion present in the right C2 nerve root (ventral side) was observed. The cervical spinal cord was significantly compressed and displaced from the right ventral side by the cyst. When cyst puncture was performed, a jelly-like content with highly viscous fluid was drained, and the capsule was removed after aspiration. However, the capsule seemed to remain in the ventral side and extend to the posterior surface of the odontoid process. We observed the ventral side and found that the dura mater was partly missing. The dura mater was coagulated and closed (Figure 5). The surgery was completed after left-sided C5-6 and C6-7 foraminotomies.

**Figure 6:**
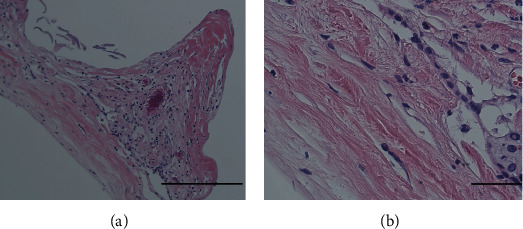
Histopathological findings. The hematoxylin and eosin staining showed synovial cells in the fibrocartilaginous capsule: (a) lower magnification; (b) higher magnification, bar: 100 *μ*m.

In the postoperative course, upper extremity pain disappeared, and left-hand grip strength was increased. Pathological examination showed that slides contained both cyst contents and normal dura mater but tumor content (Figure 6). A synovial cyst of the atlantoaxial joint was diagnosed based on preoperative imaging, operative, and pathological findings. One month after surgery, the left-hand grip strength returned to preillness strength and neuralgia disappeared. One year after surgery, the patient remained without recurrence of a synovial cyst of the atlantoaxial joint at the C1-2 level.

## 3. Discussion

Synovial cysts in the spine occur frequently in the lumbar facet joints, but synovial cysts in the atlantoaxial joint are rarely seen [1–3]. Synovial cysts of the lumbar vertebral joint often develop due to radiculopathy; however, on the other hand, synovial cysts of the atlantoaxial joint are sometimes detected accidentally or through the development of cervical myelopathy caused by a giant cyst inducing anterior compression of the cervical spinal cord. Although the etiology of the cyst has not yet been completely elucidated, it seems that chronic atlantoaxial subluxation leads to inflammatory granulation tissue or noninflammatory and degenerative fibrocartilage-like tissue, in the case of a pseudotumor lying posterior to the odontoid process. It is thought that unusual stress on the ligament caused by atlantoaxial instability generates microcracks in the ligament and results in the formation of reactive tissue and the generation of pseudotumor. C1-2 instability may essentially contribute to cyst formation and progression. Since other possibilities, such as degenerative changes in facet joints and microtrauma, may also contribute to its formation and progression, many cases associated with rheumatoid arthritis exist. However, only a few cases of patients without underlying conditions have been reported [1–3, 6].

The surgical treatment strategy for a synovial cyst of the atlantoaxial joint is controversial due to factors, such as the presence of atlantoaxial instability, level of cyst causing compression of the cervical spinal cord, severity of myelopathy, and cyst location. Previous reports include a case of cyst shrinkage after performing a posterior cervical fusion/an occipitocervical fusion on a patient associated with a synovial cyst of the atlantoaxial joint and atlantoaxial instability [1] and a case of cyst removal using various surgeries along with a posterior cervical fusion [3]. There are some other reports of cyst removal, such as a case of suboccipital craniotomy and C1-2 laminectomy [7–9], a case of transoral approach, along with an occipitocervical fixation/a C1-2 fixation [5, 10, 11], and cases of C1-2 laminectomy [11, 12] and anterolateral approach [3].

The abovementioned surgeries have different characteristics. The transoral approach seems to be a rational surgical approach, which maximally decompresses the cyst causing anterior compression of the cervical spinal cord; however, this approach is also a maximally invasive surgery. According to the study by Van Gompel et al., a transoral approach requires nasogastric tube feeding for the heal of pharyngeal wound after surgery, and the length of feeding time was 14 days on average [5]. In addition, to eliminate the postoperative instabilities of the occipital bone and the cervical spine, patients who underwent a transoral approach must receive secondary occipitocervical fusion, which makes the transoral approach more invasive.

With regard to a minimally invasive approach, there is a report of a patient with atlantoaxial instability and a relatively large synovial cyst in the atlantoaxial joint who underwent C1-2 posterior cervical fusion; as a result, the cyst regressed with immobilization, without direct removal [1].

Although all the above surgeries require an occipitocervical fusion or a posterior cervical fusion, there is a more minimally invasive approach which consists of a cyst removal through a posterior approach and one of the following surgeries: a suboccipital craniotomy alone, a cervical laminectomy alone, and a combination of these two surgeries. When synovial cysts are located lateral to the transverse ligament, a posterior approach or a posterolateral approach is often used to remove the cysts [6–9, 11].

There are further reports on a partial hemilaminectomy of the atlas to remove synovial cysts at the C1-2 level in a ventromedial region through an anterolateral approach [3, 13–15]; therefore, there are some cases without a posterior fusion.

The most minimally invasive approach is percutaneous aspiration [16]. However, because a patient treated with percutaneous aspiration may have recurrence, a surgical candidate for percutaneous aspiration seems to be the patient without a direct surgical indication. In the case of a high-surgical-risk patient, there is nothing but a wait-and-see approach, and radiological follow-up is required.

In the present study, a compressive lesion on the cervical spinal cord was not identified preoperatively as a synovial cyst. According to intraoperative and pathological findings, it seems that the compressive lesion was indeed a synovial cyst which passed through the dorsal dura. The patient did not have atlantoaxial instability. Although it was not clear if myelopathy arose from a cystic lesion, tendon reflexes of both lower extremities were enhanced and the cervical spinal cord was highly compressed. From these points of view, we determined that the patient has an increased possibility of myelopathy in the future, and therefore, we performed a resection. A kymograph did not show atlantoaxial instability, and the patient did not have underlying conditions, such as rheumatoid arthritis. The cyst expanded to the ventral side of the cervical spinal cord, but we performed a cyst removal through posterior C1-2 laminectomy because the cyst was located on the right side of the spinal cord. One year after surgery, the patient remained without recurrence of the cyst. Careful attention must be given to the presence of atlantoaxial instability in the future.

## 4. Conclusion

We experienced a case of compression of the cervical spinal cord caused by a synovial cyst of the atlantoaxial joint which passed through the dorsal dura without atlantoaxial instability. The cervical spinal cord was highly compressed, and the cyst was located on the right side of the cervical spinal cord. Therefore, we selected a relatively less invasive posterior intradural approach with C1-2 laminectomy, without posterior occipitocervical fusion. In the case of a synovial cyst without atlantoaxial instability, a resection via a posterior approach, which does not damage the structure of the atlantoaxial joint, must be one of the options, but surgical strategy should be determined after full consideration of patients' age and surgical risk.
